# Exertional heat stroke induces liver injury through the activation of the TLR4/NF-κB signaling pathway

**DOI:** 10.1016/j.bbrep.2026.102836

**Published:** 2026-10-17

**Authors:** Long Teng, Haicong Wu, Fangze Cai, Meiyan Wang, Qingqing Lin, Li Chen, Xin Chang, Dongliang Li, Xiaoling Zhou

**Affiliations:** a Fuzong Clinical Medical College, Fujian Medical University, No. 156 Xierhuan North Road, Gulou District, Fuzhou, Fujian, 350025, China; b Department of Hepatobiliary Diseases, 900th Hospital of PLA (The 900th Hospital of the Joint Logistics Support Force of PLA), No. 156 Xierhuan North Road, Gulou District, Fuzhou, Fujian, 350025, China; c Department of Gastroenterology, Central Theater General Hospital, No. 627 Wuluo Road, Wuchang District, Wuhan, Hubei, 430070, China

**Keywords:** Lipopolysaccharide, TAK-242, Intestinal barrier, Systemic inflammation, Heat stress, Endotoxemia

## Abstract

**Background:**

Exertional heat stroke (EHS) disrupts the intestinal barrier and facilitates lipopolysaccharide (LPS) translocation, thereby activating hepatic inflammatory signaling pathways and contributing to liver injury. This study aimed to investigate the role and mechanism of the Toll-like receptor 4 (TLR4)/nuclear factor kappa B (NF-κB) pathway in EHS-induced liver injury.

**Methods:**

An EHS Sprague-Dawley rat model was developed through treadmill running, with EHS defined as achieving a core temperature of 42.9°C ± 0.1°C. The model rats were pretreated with either TAK-242 (10.0 mg/kg), a TLR4 inhibitor or saline prior to running. Real-time fluorescence quantitative polymerase chain reaction (RT-qPCR), immunohistochemistry, and Western blot (WB) analyses were performed to investigate the expression of the TLR4/NF-κB pathway in the liver and the tight junction proteins in the intestine. HepG2 cells under heat stress and LPS exposure were utilized to examine TLR4/NF-κB activation at transcriptional and protein levels.

**Results:**

EHS rats exhibited significantly increased liver inflammation and elevated levels of ALT (Alanine Aminotransferase), AST (Aspartate Aminotransferase), TNF-α (Tumor Necrosis Factor-α), IL-1β (Interleukin-1β), and IL-6 (Interleukin-6) compared to the normal control group (all *P* < 0.05). There was a reduction in intestinal occludin, Zonula occludens-1 (ZO-1), E-cadherin and an increase in serum LPS levels, and upregulation of hepatic TLR4/NF-κB signaling (all *P* < 0.05). Pretreatment with TAK-242 reduced hepatic TLR4/NF-κB signaling and inflammation both in vivo and in vitro. However, it did not significantly restore occludin, ZO-1, and E-cadherin expression levels in EHS rats.

**Conclusions:**

EHS leads to liver injury through the activation of the TLR4/NF-κB signaling pathway. TAK-242 pretreatment attenuates liver inflammation by downregulating the TLR4/NF-κB signaling, supporting a mechanistic role for this pathway in EHS-induced liver injury.

## Highlights

• EHS triggers liver inflammation and gut barrier disruption.
• Gut barrier disruption elevates LPS in exertional heat stroke.
• Hepatic TLR4/NF-κB drives liver injury in EHS; TLR4 is a therapeutic target.

## 1 Introduction

Heatstroke is a life-threatening condition characterized by high mortality and morbidity, traditionally categorized into classic (non-exertional) and exertional heatstroke (EHS). Classic heatstroke primarily affects vulnerable populations, such as children, the elderly, and individuals with chronic illnesses, during exposure to high environmental temperatures [1], [2]. However, EHS typically occurs in individuals engaged in prolonged, high-intensity exertion under thermal stress, such as marathon runners, military personnel during training, and occupational laborers working in hot environments [3]. EHS is often complicated by acute kidney injury, rhabdomyolysis, hepatic impairment, or coagulopathy, which may progress to multiple organ dysfunction syndrome [4], [5]. Liver injury, a hallmark of EHS, can range from mild and self-limiting to severe acute failure [6]. Although liver function generally normalizes within 2-16 days, severe cases may involve extensive hepatocyte damage and carry a risk of fatal outcomes [7], [8]. The pathogenesis of EHS-induced liver injury is multifactorial, involving direct thermal damage, ischemic hepatitis due to systemic hypoperfusion, microvascular thrombosis, and inflammation-mediated apoptosis and necrosis [9], [10].

A key mechanistic insight involves splanchnic hypoperfusion and intestinal ischemia, which arise from heat stress-induced redistribution of blood flow. This compromise in gut perfusion promotes oxidative stress, disrupts intestinal mucosal integrity, and increases permeability to endotoxins such as lipopolysaccharide (LPS) [3]. Elevated circulating LPS levels in EHS patients correlate with the downregulation of intestinal junctional proteins, including tight junction protein occludin and the adherens junction protein E-cadherin [11], [12], [13]. Upon entering the portal circulation, LPS activates TLR4, initiating both MYD88 (myeloid differentiation primary response 88), a key adaptor protein in the TLR4 signaling pathway-dependent and independent signaling cascades. These pathways stimulate the activation of NF-κB and interferon regulatory factor, leading to a robust production of proinflammatory cytokines. Previous clinical studies have consistently observed significant elevations in IL-1β, IL-6, TNF-α, and IL-8 in EHS patients [14]. Although LPS-TLR4 signaling is well characterized in other liver injury conditions such as alcohol-associated liver disease and acute liver injury [15], [16], its specific role in EHS-induced liver injury remains unclear.

Recent studies have also highlighted peptide-based interventions as promising therapeutic candidates for inflammatory and liver injuries. Given their specificity and anti-inflammatory potential, such approaches may complement pathway-targeted strategies such as TLR4 inhibition [17], [18]. Against this background, we employed in vitro and in vivo models to investigate the role of the TLR4/NF-κB pathway in EHS-induced liver injury, with the aim of identifying potential therapeutic targets for clinical management of EHS.

## 2 Material and methods

### 2.1 Ethics statement

This study was carried out in strict accordance with the recommendations in the Guide for the Care and Use of Laboratory Animals of the National Institutes of Health. The protocol was approved by the Committee on the Ethics of Animal Experiments of the 900th Hospital of the Joint Logistics Support Force, China (approval number: 2023-44). All animals were humanely euthanized via intraperitoneal injection of sodium pentobarbital.

### 2.2 Animal models

Thirty specific-pathogen-free male Sprague-Dawley rats, aged 6 weeks and weighing 140 ± 10 g, were obtained from the Zhejiang Provincial Laboratory Animal Center (License Number: SCXK [Zhejiang] 2019-0002). The animals were housed in the Animal Experimentation Center of the 900th Hospital under controlled conditions including a 12-h light/dark cycle, an ambient temperature of 25°C, and a relative humidity of 55%; food and water were provided ad libitum.

After 7 days of acclimatization, the rats underwent treadmill adaptation training for 7 days, starting at 10 m/min for 10 min and gradually increasing to 20 m/min for 30 min by day 5, followed by a 2-day recovery period. Six rats failed to adapt or sustained injuries and were excluded. Therefore, 24 rats were included in the formal experiments. These rats were used in two experimental designs. Design 1 included 6 rats divided into NC and EHS groups (n = 3 per group) for model validation. Design 2 included the remaining 18 rats, randomly assigned to NC, EHS, and EHS + TAK-242 groups (n = 6 per group) for the main analyses. Within Design 2, a subgroup of 9 rats (n = 3 per group) was used for portal vein LPS measurement. This subgroup was not additional animals, but part of the 18 rats in Design 2.

On day 8, rats in the EHS + TAK-242 group received TAK-242 (10.0 mg/kg, intraperitoneally) before exercise [19], whereas rats in the NC and EHS groups received an equivalent volume of saline. The EHS and EHS + TAK-242 groups ran at 20 m/min for 2 h under heat and humidity stress (35°C ± 2°C and 80% ± 5% relative humidity). Core temperature was monitored at 15-min intervals using a rectal digital thermometer (Digi-Sense, 91428-05), with the treadmill briefly paused for each measurement [20]. EHS was defined as a core temperature of 42.9°C ± 0.1°C immediately after exercise, as shown in Fig. S1. All rats in the EHS and EHS + TAK-242 groups reached this predefined criterion. Six hours after exercise, the rats were euthanized, and samples were collected for subsequent analyses. This protocol reliably induced severe hyperthermia, analogous to the physiological strain experienced during prolonged, high-intensity exertion in hot environments [21], [22].

### 2.3 FITC-dextran flux assay

Intestinal barrier permeability was measured using fluorescein isothiocyanate-dextran (FITC-dextran, Sigma–Aldrich, USA, 46944). On the day of sacrifice, rats were deprived of food for 4 h, and then the FITC-dextran tracer (4 kDa, 0.6 mg/g body weight) was administered intragastrically by oral gavage using a stainless-steel feeding needle, in a total volume of 0.1 ml PBS. After 3 h, blood was collected via cardiac puncture (approx. 2 ml) into serum separator tubes to obtain hemolysis-free serum, and this procedure was carried out prior to euthanasia. Measure intestinal permeability, which correlates with fluorescence intensity of appropriately diluted serum, using a Hitachi F-4500 fluorescence spectrophotometer (excitation of 488 nm and an emission of 520 nm). A standard curve for FITC-dextran was prepared by serially diluting a known amount of FITC-dextran in rats’ serum.

### 2.4 Measurement of intestinal oxidative stress markers

Ileal tissues were homogenized in ice-cold PBS and centrifuged to obtain the supernatant. The levels of malondialdehyde (MDA) and superoxide dismutase (SOD) in intestinal tissue were measured using commercially available assay kits according to the manufacturer's instructions. The results were normalized to total protein content and expressed as MDA and SOD levels per milligram of protein.

### 2.5 Sampling and storage

Blood was collected via cardiac puncture (approx. 5 ml) prior to euthanasia, anticoagulated with lithium heparin, and centrifuged at 2,500 rpm for 10 min. The plasma was subsequently stored at −80°C. Liver and intestinal tissues were flash-frozen in liquid nitrogen and stored at −80°C. Additionally, the left lateral lobe of the liver and ileum tissues were fixed in 10% neutral buffered formalin for 24 h and then paraffin-embedded for histological examination.

### 2.6 Liver function tests

Serum levels of alanine aminotransferase (ALT) (C009–2, Nanjing Jian Cheng Biological Co., Ltd., Nanjing, China) and aspartate aminotransferase (AST) (C010–2, Nanjing Jian Cheng Biological Co., Ltd., Nanjing, China) were quantified using commercially available diagnostic kits, following the manufacturer's instructions.

### 2.7 Cytokine measurements

The levels of TNF-α, IL-1β, IL-6, and LPS in plasma were measured using an Enzyme-Linked Immunosorbent Assay (ELISA). Commercially available kits were utilized in accordance with the manufacturer's instructions (R&D Systems, Minneapolis, MN, USA): TNF-α (RTA00), IL-1β (RLB00), IL-6 (R6000B), and LPS (Elabscience, Houston, TX, USA, E-EL-0180).

### 2.8 Histological analysis of necrosis

Intestinal and liver tissues were fixed in 4% paraformaldehyde for 96 h, embedded in paraffin, sectioned to a thickness of 3 μm, and stained with hematoxylin and eosin (H&E). Pathological evaluation and imaging were conducted at magnifications of 20× and 50×. The scoring of ileum and liver damage was performed based on established criteria [23], [24]. Histological scoring was performed independently by two blinded investigators, and the mean value was used for statistical analysis.

### 2.9 Cell culture and pretreatment

HepG2 cells (a liver cancer cell line, Procell, CL-0103) and AML-12 cells (Procell, CL-0601) were cultured in Dulbecco's Modified Eagle's Medium (DMEM, Gibco, USA, C11995500BT) supplemented with 10% fetal bovine serum (Gibco, Australia, 10099141) at 37°C in a humidified atmosphere containing 5% CO^2^. Both cell lines were authenticated by short tandem repeat (STR) profiling before use, and the authentication reports were provided as supplementary materials. The experimental groups were categorized as follows: (1) NC group: cultured at 37°C; (2) TAK-242 + HS group: pretreated with 1 μM TAK-242 (MCE, USA, HY-11109) for 12 h, followed by exposure to 43°C for 2 h, and then recovered at 37°C for 3 h [25]; (3) HS group: heat-treated as described above without pretreatment with TAK-242; (4) LPS + HS group: pretreated with 100 ng/ml LPS (MCE, USA, HY-D1056) for 24 h prior to heat exposure [26]; (5) TAK-242 + LPS + HS group: pretreated with both TAK-242 and LPS before heat exposure. Supernatants and cells were collected for subsequent analysis. A 43°C heat-stress exposure for 2 h was selected based on established in vitro heat-stress protocols to induce a robust cellular heat-shock response. Cells were subsequently returned to 37°C for 3 h to assess post-heat-stress recovery responses, which are relevant to the persistent cellular responses observed after exertional heat stroke [27].

### 2.10 Real-time fluorescence quantitative polymerase chain reaction (RT-qPCR)

Total RNA was extracted using the RNeasy Mini Kit (Qiagen, Hilden, Germany, 74134). cDNA synthesis was conducted with the PrimerScript RT Kit (Takara, Japan, RR037A). Amplification was performed using the CFX96 Real-Time System (Bio-Rad, USA, 1845096). mRNA expression levels were quantified using the 2^−ΔΔ^CT method. The primer sequences utilized in this study are as follows:

(Rat) TLR4 forward: 5’ - AGACCTGTCCCTGAACCCTAT - 3’;

TLR4 reverse: 5’ - CGATGGACTTCTAAACCAGCCA - 3’;

(Rat) E-cadherin forward: 5’ - AAAGACCAAGAGCAGGAGTGTG - 3’;

E-cadherin reverse: 5’ - TGAGGGTACATGTCGGGGAG - 3’;

(Rat) Occludin forward: 5′- GGCAATCCGGTTTTATGCTG - 3’;

Occludin reverse: 5′- TGAGGGTACATGTCGGGGAG - 3’;

(Rat) GAPDH forward: 5’ - GGAGCGAGATCCCTCCAAAAT - 3’;

GAPDH reverse: 5’ - GGCTGTTGTCATACTTCTCATGG - 3’.

(Human) TLR4 forward: 5’ - CAACAAAGGTGGGAATGCTT - 3’;

TLR4 reverse: 5’ - TGCCATTGA AAGCAACTCTG - 3’;

(Human) GAPDH forward: 5’ - GAAGGTGAAGGTCGGAGTC - 3’;

GAPDH reverse: 5’ -GAAGATGGTGATGGGATTTC - 3’.

### 2.11 Western blotting (WB)

Total protein from intestinal tissues, liver tissues, and HepG2 and AML-12 cells were extracted using ice-cold RIPA lysis buffer (Beyotime, P0013B) supplemented with protease inhibitor cocktail (Beyotime, P1005) and phosphatase inhibitor cocktail (Beyotime, P1082) at a volume ratio of 100:1:1. The samples were then milled and lysed on ice for 35 min, followed by centrifugation for 10 min at 12,000 rpm to collect the supernatant for total protein extraction. A BCA Kit (Beyotime, P0010) was employed to determine the protein concentration. Following SDS-PAGE gel electrophoresis, the proteins were transferred to a PVDF membrane (Millipore, USA, IPVH00010). The membrane was blocked for 1 h with 5% skimmed milk and subsequently incubated with primary antibodies: anti-TLR4 (1:1000, Abcam, USA, ab13867), anti-NF-κB (1:1000, CST, 8242S), anti-p-NF-κB (1:1000, CST, 3033S), anti-IKKα+β (1:1000, Abcam, USA, ab178870), anti-Ikkα (1:1000, Abcam, USA, ab38515), anti-MyD88 (1:1000, Abcam, USA, ab219413), anti-E Cadherin (1:1000, Abcam, USA, ab231303), anti-occludin (1:1000, Abcam, USA, ab216327), and anti-GAPDH (1:1000, CST, 2118). The incubation was performed overnight at 4°C. The following day, the PVDF membranes were incubated with Goat anti-Rabbit IgG HRP (1:5000, Affinity, Cincinnati, OH, S0001) for 1 h. Finally, the membranes were immersed in ECL chemiluminescent solution (Biosharp, China, BL520A) and imaged using the ChemiDoc imaging system (Bio-Rad, USA, 12003154). To analyze the expression of target proteins, the optical density was normalized to the GAPDH level using ImageJ 64-bit Java 1.8.0 (NIH).

### 2.12 Immunohistochemistry (IHC) and immunofluorescence (IF)

Formalin-fixed sections of the ileum and liver were dewaxed, rehydrated, and subjected to antigen retrieval in citrate buffer. IHC was performed using antibodies anti-TLR4 (1:1000, Abcam, USA, ab13867), anti-E Cadherin (1:1000, Abcam, USA, ab231303), and anti-occludin (1:1000, Abcam, USA, ab216327). For IF, a triple-labeling technique was employed to visualize E-cadherin, occludin, and ZO-1. Besides, staining of TLR4 in HepG2 cells was performed using an anti-TLR4 primary antibody. Images were captured using an Olympus microscope and quantified with ImageJ, based on the average optical density (fold change compared to the negative control).

### 2.13 Statistical analysis

All statistical analyses were conducted using GraphPad Prism (version 9, San Diego, USA). Data normality was assessed using the Shapiro-Wilk test, and all datasets were confirmed to meet the normality assumption prior to applying parametric statistical tests (e.g., Student's t-test or one-way ANOVA). Differences were considered statistically significant at *P* < 0.05. All experiments were replicated at least three times to ensure reliability.

## 3 Results

### 3.1 EHS induces liver injury and upregulates hepatic TLR4 expression in rat models

To elucidate the role of the TLR4/NF-κB signaling pathway in EHS, we developed a rat model by treadmill running under high ambient temperature and humidity (Fig. 1A–B). Core body temperature was continuously monitored, with EHS defined as achieving a core temperature of 42.9°C ± 0.1°C. Histological examination of liver tissues from control animals revealed well-preserved architecture without significant pathological abnormalities. In comparison to the NC group, EHS rats exhibited extensive structural liver damage and inflammatory infiltration (Fig. 1C). Serum levels of ALT and AST were significantly elevated in the EHS group (Fig. 1D, all *P* < 0.01). Furthermore, transcriptional upregulation of TLR4 was evident in EHS liver tissues, with mRNA expression significantly increased relative to the NC group (Fig. 1E, *P* < 0.01). IHC analysis also demonstrated a substantial enhancement of TLR4 expression at the protein level in the EHS group (Fig. 1F, *P* < 0.01). These results indicated that EHS induces liver injury and upregulates hepatic TLR4 expression.

**Fig. 1:**
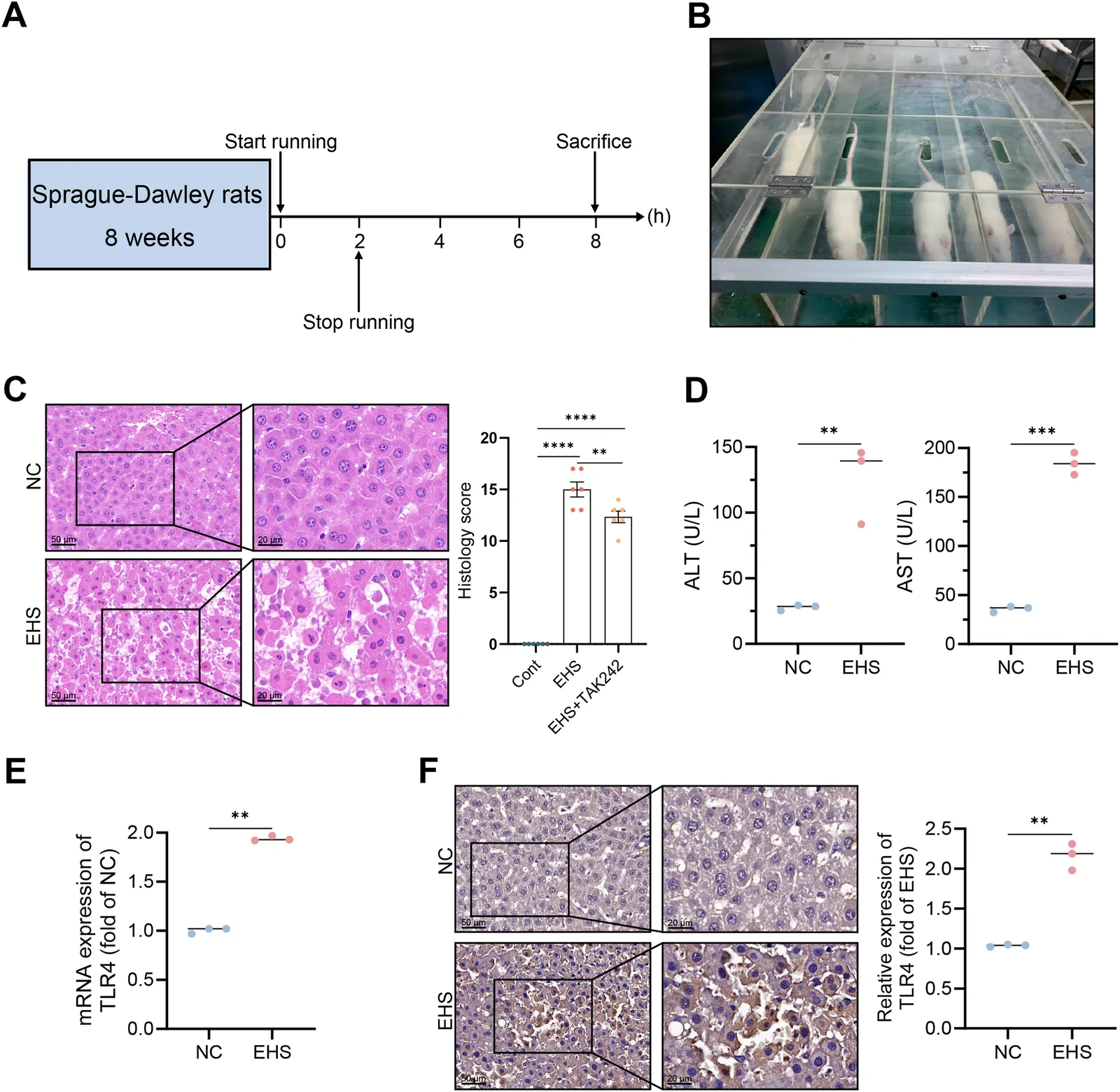
EHS induces liver injury and upregulates hepatic TLR4 expression in rats. (A, B) Schematic timeline of the EHS rat model. (C) Representative H&E-stained liver sections (scale bars = 100 μm) and quantitative histopathological scores of liver injury. (D) Serum levels of AST and ALT in the NC and EHS groups. (E) Hepatic TLR4 mRNA expression in the NC and EHS groups. (F) Immunohistochemical detection of TLR4 protein, which was predominantly localized in hepatocytes. Data are presented as mean ± standard deviation (n = 3 per group). *P < 0.05, **P < 0.01, ***P < 0.001. Abbreviations: NC, normal control; EHS, exertional heat stroke; AST, aspartate transaminase; ALT, alanine aminotransferase; TLR4, toll like receptor 4.

### 3.2 TLR4 inhibition attenuates EHS-induced liver injury and TLR4/NF-κB signaling activation

To further elucidate the role of TLR4 in EHS-induced liver injury, we evaluated the effects of TAK-242 pretreatment on liver injury and TLR4 pathway activation (Fig. 2A). Histological examination of liver tissues revealed that EHS-induced substantial tissue damage and inflammatory cell infiltration were markedly attenuated following TAK-242 administration (Fig. 2B). Serum levels of ALT, AST, and pro-inflammatory cytokines were significantly decreased in the EHS + TAK-242 group compared to the EHS group (Fig. 2C–D, all *P* < 0.05). At the molecular level, the EHS-induced upregulation of TLR4 mRNA expression in liver tissue was significantly reversed by TAK-242 pretreatment (Fig. 2E, all *P* < 0.05). IHC analysis further confirmed that TAK-242 reduced EHS-induced TLR4 protein expression (Fig. 2F, *P* < 0.05). Moreover, WB analysis showed that TAK-242 pretreatment effectively attenuated the EHS-induced upregulation of TLR4, IKK-α, phospho-NF-κB and Myd-88 protein levels (Fig. 2G–H, all *P* < 0.05).

**Fig. 2:**
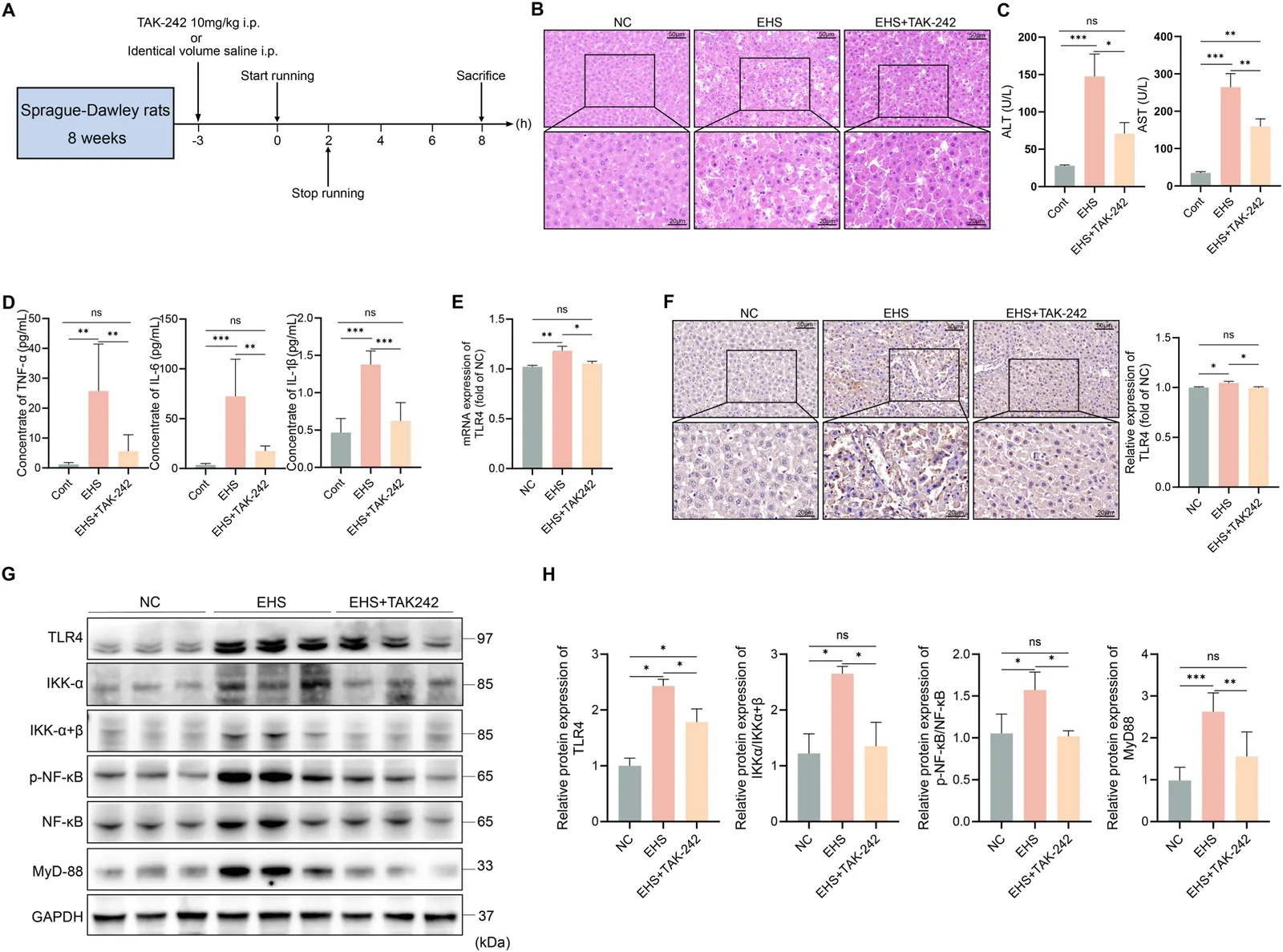
**TAK-242 attenuates EHS-induced hepatic inflammation and suppresses TLR4/NF-κB signaling activation.** (A) Experimental timeline showing TAK-242 (10.0 mg/kg, i.p.) or saline pretreatment followed by EHS induction. (B) Representative H&E-stained liver sections. Scale bars: 50 μm and 20 μm. (C) Serum AST and ALT levels. (D) Serum TNF-α, IL-6, and IL-1β levels. (E) Hepatic TLR4 mRNA expression. (F) Immunohistochemical staining of TLR4 in liver tissues. Scale bars: 50 μm and 20 μm. (G) Western blot analysis of TLR4/NF-κB signaling pathway proteins. GAPDH was used as a loading control. (H) Quantitative analysis of TLR4, IKK-α, IKK-α+β, p-NF-κB/NF-κB, and MyD88 protein expression. Data are presented as mean ± standard deviation (n = 6 per group). *P < 0.05, **P < 0.01, ***P < 0.001. Abbreviations: EHS, exertional heat stroke; ALT, alanine aminotransferase; AST, aspartate transaminase; TNF-α, tumor necrosis factor-α; IL-6, interleukin-6; IL-1β, interleukin-1β; IHC, immunohistochemistry; NF-κB, nuclear transcription factor kappa-B.

These findings suggested that TAK-242 suppressed EHS-induced upregulation of TLR4 transcription and effectively inhibited the activation TLR4/NF-κB signaling, thereby ameliorating liver injury.

### 3.3 TAK-242 suppresses heat- and LPS-induced TLR4 signaling in hepatocytes

To investigate the cell-autonomous role of TLR4 in the hepatic heat stress response, we established an in vitro model of heat stroke using HepG2 and AML-12 cells and conducted various group pretreatments (Fig. 3A). Q-PCR analysis demonstrated that heat stress with and without LPS pretreatments significantly increased TLR4 mRNA levels compared to the NC group (all *P* < 0.05). TAK-242 pretreatment effectively mitigated this upregulation, showing significantly lower TLR4 expression in the TAK-242 + heat stroke and TAK-242 + LPS + heat stroke groups compared to the heat stroke and LPS + heat stroke groups, respectively (Fig. 3B, all *P* < 0.05). A consistent trend was observed in HepG2 cells by immunofluorescence, where both heat stress conditions also markedly elevated TLR4 protein levels. Similarly, this increase was suppressed by TAK-242, with treated groups showing significantly lower fluorescence intensity (Fig. S2, all *P* < 0.05). Furthermore, WB analysis indicated that the protein levels of key components of the TLR4 signaling pathway—including TLR4, IKK-α, phosphorylated NF-κB, and the adaptor protein MyD88—were markedly elevated following heat stress both in HepG2 and AML-12 cells. This elevation was further enhanced by LPS co-stimulation; however, TAK-242 pretreatment significantly reduced the activation of this pathway under both heat stress and LPS challenge conditions (Fig. 3C–D, Fig. S3, all *P* < 0.05). Additionally, heat stroke and LPS + heat stroke pretreatments facilitated the nuclear translocation of NF-κB, which was inhibited by TAK-242 pretreatment (Fig. 3E–F, all *P* < 0.05).

**Fig. 3:**
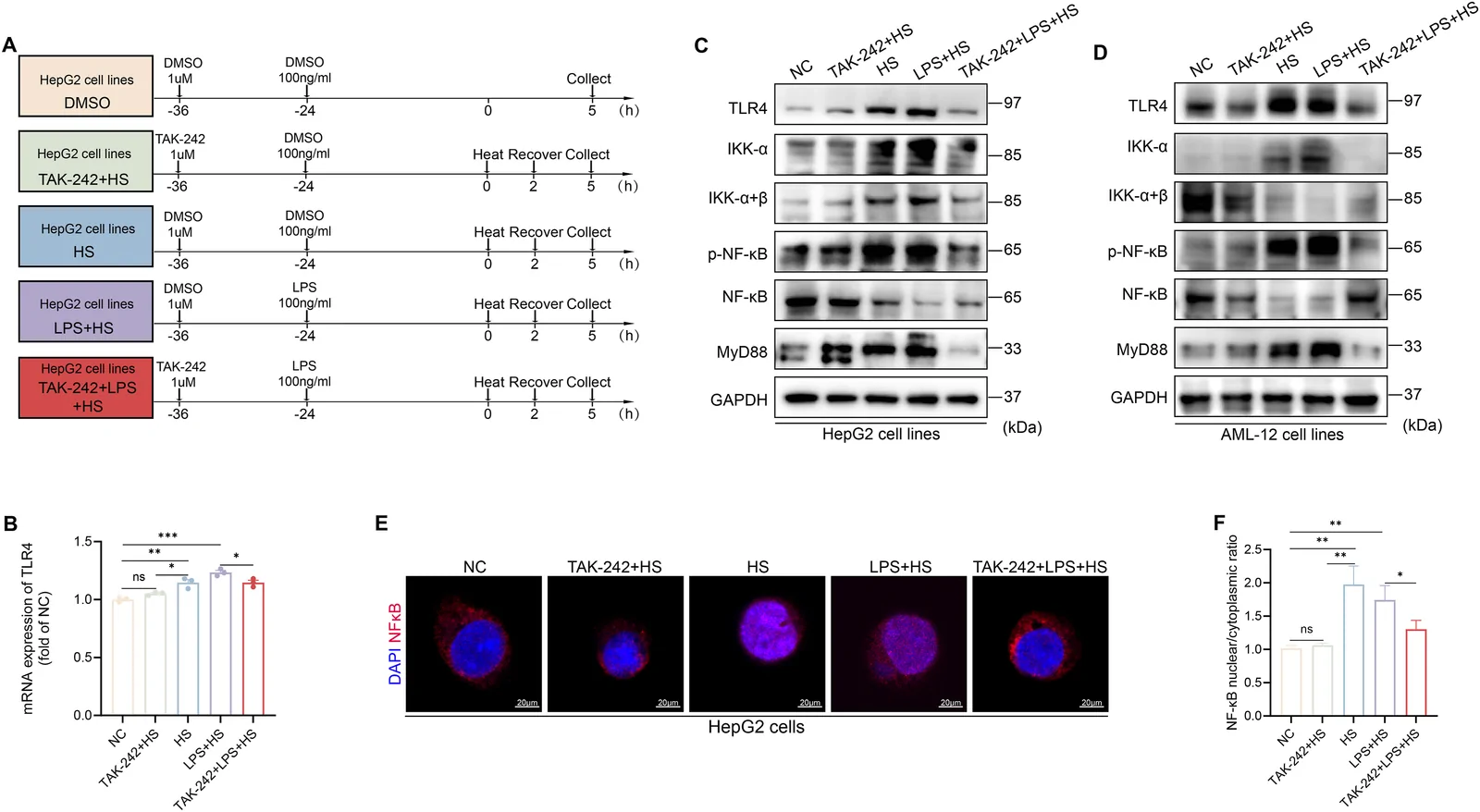
TAK-242 inhibits heat- and LPS-induced TLR4 downstream signaling in HepG2 and AML-12 cells. (A) Experimental timeline of the in vitro heat stress model. Cells were pretreated with TAK-242 (1 μM) or vehicle, stimulated with LPS (100 ng/mL) where indicated, exposed to heat stress (43°C for 2 h), and recovered at 37°C before harvesting. (B) TLR4 mRNA expression levels under the indicated conditions, as determined by qRT-PCR. (C, D) Western blot analysis of TLR4 signaling pathway proteins in HepG2 cells (C) and AML-12 cells (D). GAPDH was used as a loading control. (E, F) Representative immunofluorescence staining of NF-κB in the indicated groups. Images were acquired from three randomly selected fields in three independent samples per group. Data are presented as mean ± standard deviation from three independent experiments (n = 3 per group). *P < 0.05, **P < 0.01, ***P < 0.001. Abbreviations: LPS, lipopolysaccharide; NF-κB, nuclear transcription factor kappa-B.

These in vitro results indicated that TAK-242 primarily acted on the post-transcriptional level to inhibit TLR4-mediated pro-inflammatory signaling in hepatocytes under the combination of heat stroke and endotoxic stress.

### 3.4 EHS induced intestinal barrier dysfunction and elevated systemic LPS levels

To assess the impact of TLR4 inhibition on intestinal barrier function during EHS, rats were administered TAK-242 prior to EHS induction. Histological analysis revealed that EHS induced severe intestinal mucosal injury and inflammatory infiltration; however, this injury was not reversed by the administration of TAK-242 (Fig. 4A). Serum LPS levels were significantly elevated in the EHS group compared to the NC group, and TAK-242 pretreatment did not lead to a significant improvement in LPS levels. Importantly, changes in portal vein LPS were consistent with those seen in serum LPS (Fig. 4B, Fig. S4). At the transcriptional level, EHS significantly downregulated the expression of E-cadherin (adherens junction) and occludin (tight junction) compared to the NC group, and this reduction was not reversed by TAK-242 pretreatment (Fig. 4C). WB analyses further demonstrated that TAK-242 failed to prevent EHS-induced loss of the tight junction protein occludin and adherens junction protein E-cadherin in intestinal tissues (Fig. 4D). Consistent with these findings, IF and IHC staining revealed a persistent disruption of the epithelial junction architecture, with no significant restoration of E-cadherin or occluding, ZO-1 expression in EHS rats pretreated with TAK-242 (Fig. 4, Fig. 5). Correspondingly, intestinal barrier function was impaired, as evidenced by a significant increase in FITC-dextran permeability, which also remained unaffected by TAK-242 pretreatment (Fig. 4F). In addition, EHS markedly increased intestinal MDA levels and reduced SOD activity, while TAK-242 showed no significant effect on either marker. These results suggest that persistent oxidative stress may be one of the mechanisms underlying intestinal barrier disruption in EHS (Fig. 4G).

**Fig. 4:**
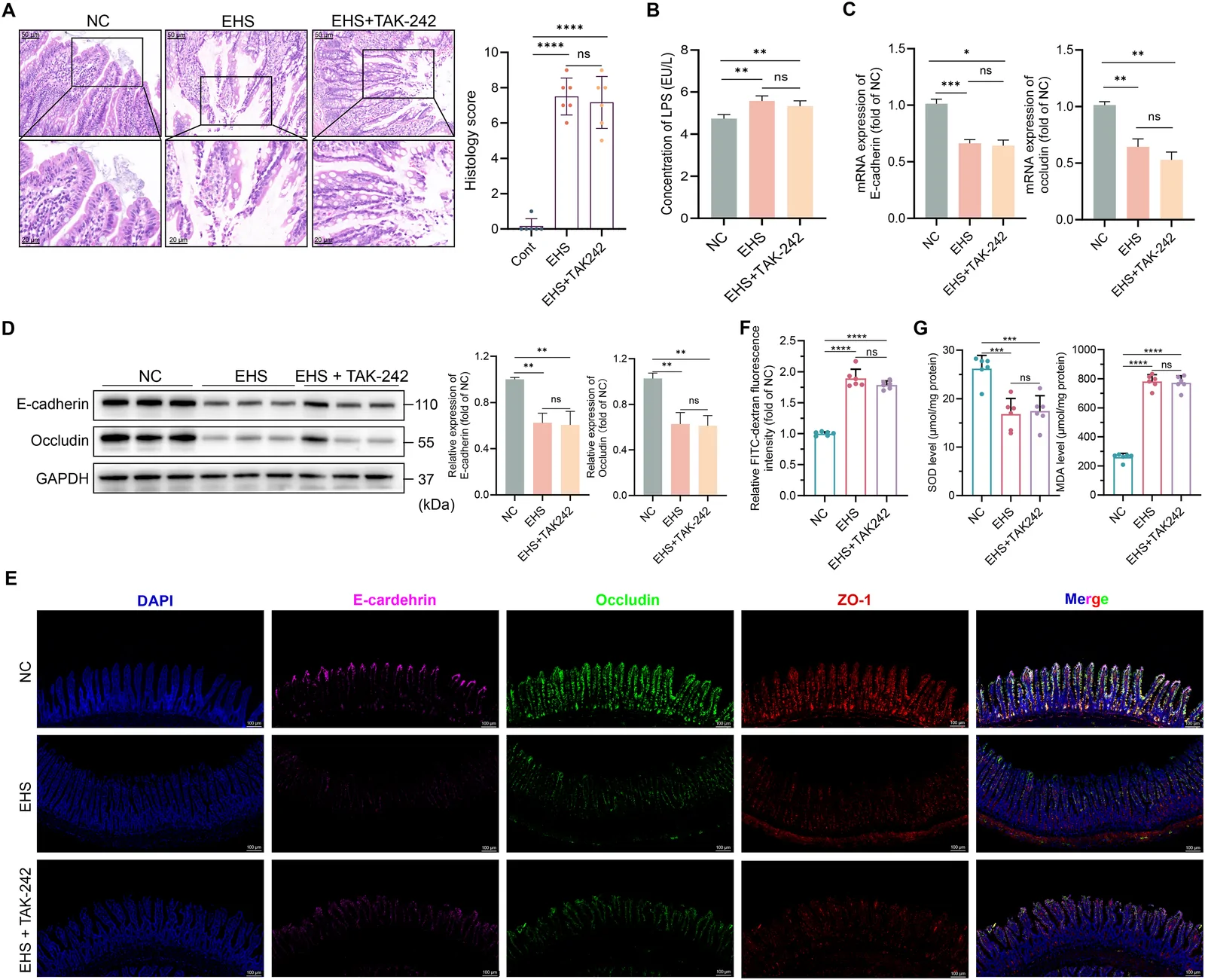
EHS induces intestinal barrier disruption and elevated systemic LPS (A) Representative H&E-stained intestinal sections and quantitative histopathological scores for intestinal injury. Scale bars: 50 μm and 20 μm. (B) Serum LPS concentrations in the NC, EHS, and EHS + TAK-242 groups. (C) Intestinal E-cadherin and occludin mRNA levels. (D) Western blot analysis of E-cadherin and occludin protein expression. GAPDH was used as a loading control. (E) Immunofluorescence staining of E-cadherin (pink), occludin (green), and ZO-1 (red). Nuclei were stained with DAPI (blue). Scale bar: 50 μm. (F) FITC-dextran permeability in rats. (G) SOD activity and MDA levels in intestinal tissues of the NC, EHS, and EHS + TAK-242 groups. Data are presented as mean ± standard deviation (n = 6 per group). *P < 0.05, **P < 0.01, ***P < 0.001. Abbreviations: NC, normal control; EHS, exertional heat stroke; LPS, lipopolysaccharide.

**Fig. 5:**
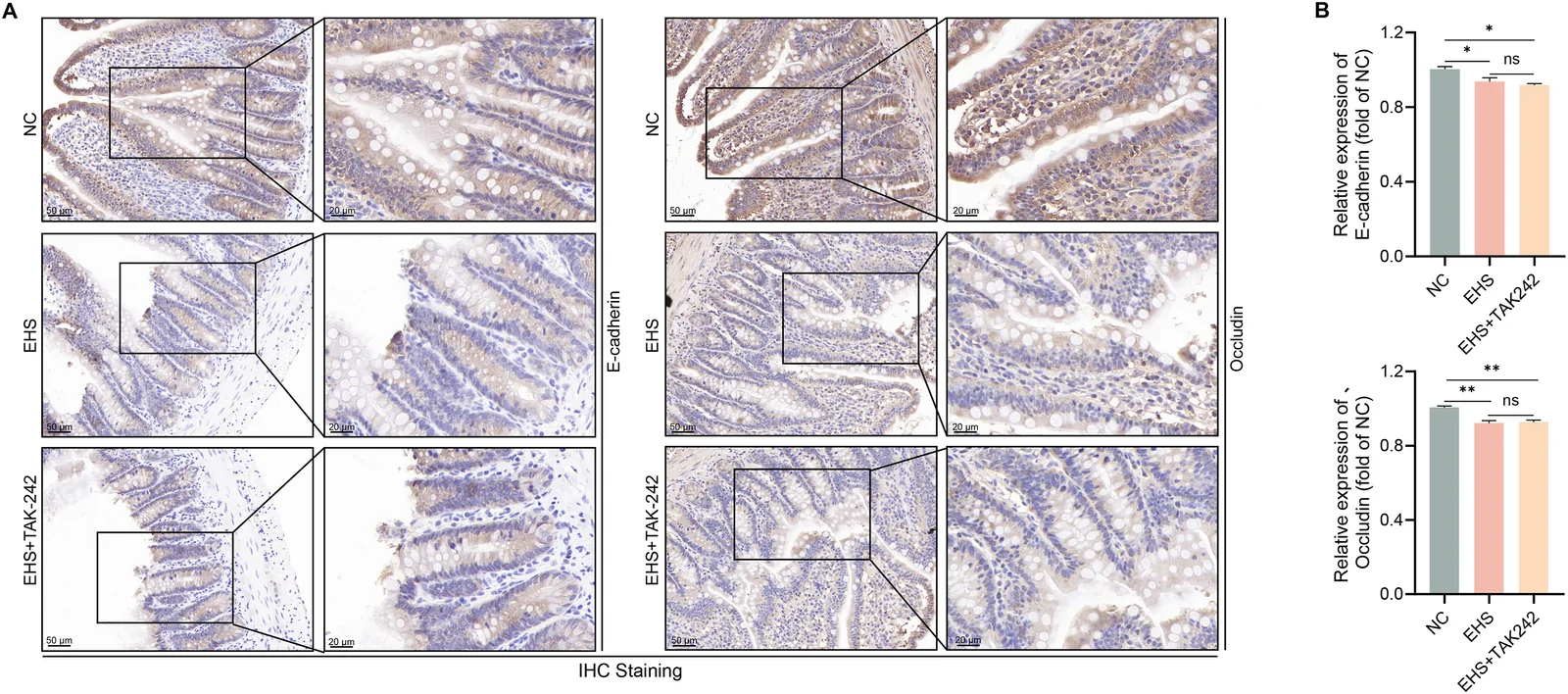
Expression of E-cadherin and occludin across different groups (A) Representative IHC staining of E-cadherin and occludin in the NC, EHS, and EHS + TAK-242 groups. Scale bars: 50 μm and 20 μm. (B) Quantitative analysis of IHC staining for E-cadherin and occludin. Data are presented as mean ± standard deviation (n = 6 per group). *P < 0.05, **P < 0.01. Abbreviations: NC, normal control; EHS, exertional heat stroke.

These findings suggest that EHS induced intestinal barrier dysfunction and elevated systemic LPS levels. However, the pharmacological inhibition of TLR4 with TAK-242 alone was not sufficient to preserve intestinal barrier function and prevent endotoxin translocation in EHS.

## 4 Discussion

The present study demonstrated that EHS leads to liver injury, loss of intestinal tight junction proteins and adherens junction protein (occludin and E-cadherin), elevated systemic LPS levels, and activation of hepatic TLR4/NF-κB signaling pathway. Importantly, TLR4 pretreatment attenuated inhibition with TAK-242 effectively attenuates hepatic TLR4/NF-κB activation and liver injury, but did not restore the intestinal barrier function. These findings support a mechanistic role for hepatic TLR4 signaling in EHS-induced liver injury.

Consistent with our findings, previous studies conducted in both in vivo and in vitro models have reported that the hepatoprotective effects of TAK-242 are mediated through the enhancement of cell-autonomous inhibition of TLR4 signaling in hepatocytes, rather than through the improvement of intestinal barrier function [28], [29]. Current treatment strategies for EHS primarily focus on rapid cooling and supportive care, with limited options for targeted pharmacological intervention [30], [31]. Emerging pharmacological targets for EHS include downstream effectors such as the NLRP3 inflammasome and mitochondrial ROS; in contrast, TLR4 inhibition acts upstream to broadly suppress innate immune activation, offering a strategic intervention point [32]. Our studies further demonstrate that TAK-242 directly inhibited heat- and LPS-induced TLR4 activation in hepatocytes, resulting in reduced expression of TLR4, IKK-α, phospho-NF-κB, and MyD88, as well as preventing NF-κB nuclear translocation. TLR4 inhibition is found to protect against EHS-induced liver injury, even under conditions of persistent endotoxemia. The inhibitor TAK-242 exerts its most pronounced effects at the protein and signaling levels. Furthermore, the cellular mechanisms underlying TAK-242's action appears to primarily involve post-transcriptional regulation. Specifically, EHS significantly increases hepatic TLR4 expression, an effect that is partially attenuated by TAK-242 pretreatment. TAK-242 significantly reduces the EHS-induced upregulation of TLR4, phospho-NF-κB, and downstream inflammatory mediators, despite only partially normalizing TLR4 transcript levels. This differential effect implies that TAK-242 may facilitate the degradation of TLR4 protein, inhibit its trafficking to the cell membrane, or interfere with downstream signaling components such as MyD88 or NF-κB [33], [34]. Even under conditions of combined heat and LPS stress, the capacity to suppress NF-κB nuclear translocation remains, supporting a mechanism that operates beyond TLR4 gene transcription. These findings provide mechanistic insights into TAK-242-mediated TLR4 inhibition in EHS-induced liver injury.

Regarding the relationship between changes in TAK-242 and intestinal barrier integrity, the current study shows that TAK-242 did not restore intestinal barrier integrity. This conclusion is supported by the persistent downregulation of both the E-cadherin (adherens junction) and occludin (tight junction), ongoing mucosal damage, and the unimproved epithelial junction architecture. These findings strongly suggest that TLR4 signaling is not the primary driver of gut barrier disruption during EHS. Other mechanisms, such as direct thermal injury, ischemia-reperfusion damage, redox imbalance, or activation of alternative inflammatory pathways (e.g., NLRP3 inflammasome or MAPK signaling), may play more central roles in intestinal pathology [35], [36]. This interpretation is consistent with clinical observations that EHS patients often exhibit prolonged gastrointestinal dysfunction resistant to conventional supportive treatments [37]. Furthermore, TAK-242 did not significantly reduce endotoxemia, as indicated by unchanged serum and portal vein LPS levels compared with the EHS-only group. TLR4 is known to play a crucial role in maintaining intestinal immune homeostasis and facilitating the clearance of translocated pathogens [38]. Its inhibition may compromise bacterial phagocytosis by mucosal immune cells, impair Kupffer cell-dependent LPS clearance in the liver, or alter gut microbiota composition, any of which could perpetuate endotoxemia [38], [39]. Alternatively, TAK-242 may affect non-TLR4 receptors involved in bacterial recognition or LPS detoxification [40]. Further studies are necessary to explore these possibilities, including assessments of bacterial translocation rates, phagocytic function, and microbial shifts following TLR4 inhibition. Importantly, the persistent endotoxemia observed after TAK-242 pretreatment indicates that hepatic TLR4 inhibition alone is insufficient to correct gut barrier dysfunction in EHS.

Several limitations of our study warrant acknowledgment. Firstly, although HepG2 and AML-12 cells facilitated mechanistic investigation of hepatocyte responses, they do not fully recapitulate primary hepatocytes or the multicellular hepatic microenvironment, including the contribution of non-parenchymal cells such as Kupffer cells, hepatic stellate cells, and liver sinusoidal endothelial cells. Secondly, although our primary focus was on TLR4 signaling, TLR2 and the NLRP3 inflammasome may also contribute to EHS-induced liver injury [32]. Thirdly, we did not directly assess the phosphorylation status of individual IKK subunits; therefore, future studies should examine phospho-IKKα/β to more precisely define upstream activation of the canonical NF-κB pathway in EHS-induced liver injury. Fourthly, although TAK-242 pretreatment provided functional evidence that TLR4/NF-κB signaling contributes to EHS-induced liver injury, it did not improve intestinal barrier disruption or circulating LPS levels, suggesting that direct heat injury or endogenous danger signals may also be involved and that the causal link within the gut–liver axis remains incomplete. Further studies using LPS neutralization, barrier-protective interventions, and genetic or cell-specific TLR4 inhibition are warranted. Fifthly, TAK-242 was used primarily as a mechanistic inhibitor and was administered before EHS induction; therefore, the present study does not address post-onset treatment efficacy. Future studies should use a post-insult design if TAK-242 is further developed for therapeutic use. Sixthly, the absence of exercise-only, heat-only, TAK-242-only, and vehicle-control groups limits interpretation of the individual effects of exercise, heat stress, and TAK-242. Future studies should include these controls. Seventhly, the present study used male rats to control for estrous-cycle variability and to align with the higher clinical severity of EHS in males [41], thereby focusing the mechanistic analysis on TLR4-mediated injury. Future direct comparisons of both sexes are needed to evaluate any sexual dimorphism in therapeutic response.

Given these limitations, several directions for future research are warranted. First, future studies should employ a post-insult administration model to evaluate the true therapeutic potential of TLR4 blockade in EHS, better reflecting real-world clinical scenarios. Second, combination therapies targeting intestinal barrier protection and hepatic inflammation, including longitudinal assessment of barrier integrity during heat stress and evaluation of heat shock proteins as mechanistic mediators. Third, single-cell or immunofluorescence studies are needed to delineate the cell-specific roles of TLR4 signaling in EHS-induced liver injury, consistent with recent single-cell transcriptome evidence demonstrating heat stroke–associated immune injury in the liver and kidney [5]. In addition, some analyses were performed with a limited sample size, particularly the initial model-validation experiment and portal vein LPS measurement; therefore, future studies with larger cohorts are needed to further validate these findings.

In conclusion, this study suggests that activation of the LPS-TLR4-NF-κB signaling pathway contributes to EHS-induced liver injury. TAK-242 pretreatment attenuated liver injury, even in the presence of persistent intestinal barrier dysfunction. These findings offer new insights into the mechanisms underlying EHS-induced organ damage and support further investigation of TLR4 signaling as a mechanistic pathway in EHS-induced liver injury.

## Ethics declaration

This study was conducted in accordance with the ARRIVE (Animal Research: Reporting of In Vivo Experiments) guidelines. This study was approved by the the Committee on the Ethics of Animal Experiments of the 900th Hospital of the Joint Logistics Support Force, China. (Approval No. approval number: 2023-44)

## Patient consent for publication

Not applicable. The study does not involve human participants, and therefore no patient consent for publication is required.

## Availability of data and materials

All data generated or analyzed during this study are included in this published article and its supplementary information files.

## Ethics approval and consent to participate

This study was carried out in strict accordance with the recommendations in the Guide for the Care and Use of Laboratory Animals of the National Institutes of Health. The protocol was approved by the Committee on the Ethics of Animal Experiments of the 900th Hospital of the Joint Logistics Support Force, China (approval number: 2023-44). All animals were humanely euthanized via intraperitoneal injection of sodium pentobarbital.

## Funding

This work was supported by from 10.13039/501100003392Natural Science Foundation of Fujian Province of China (2023J011343), 900th Hospital of the Joint Logistic Support Force Fund (Grant Number: 2021JQ04), 10.13039/501100003392Natural Science Foundation of Fujian Province of China (2021J011263), and Fujian University of Traditional Chinese Medicine's School-Managed Project (XB2023178).

## Declaration of competing interest

All authors have completed the ICMJE uniform disclosure form. The authors have no conflicts of interest to declare.

## Data Availability

Data will be made available on request.
