# Supplementary material for: Exertional heat stroke induces liver injury through the activation of the TLR4/NF-κB signaling pathway
Source: Biochem Biophys Rep. 2026 Oct 17;48:102836. doi: 10.1016/j.bbrep.2026.102836 (PMC13696764; doi:10.1016/j.bbrep.2026.102836)
Supplement: Supplementary file 1 [file mmc2.pdf]

Hep G2细胞STR鉴定报告

一、 材料处理和检验方法

取适量Hep G2细胞(编号PC-H2024042816,1×10^6)使用TIANamp Genomic DNA Kit提取DNA，采用Microreader™21 ID System扩增20个STR位点和性别鉴定位点，使用智阅基因分析仪GenReader 7010进行PCR产物检测，使用GeneMapper Software6软件（Applied Biosystems）对检测结果进行分析，并与ExPASy数据库进行比对。

二、 检测结果

实验中阴性及阳性对照结果均正确。

Hep G2 细胞株的 STR 位点和 Amelogenin 位点的基因分型结果见附表，分型图谱见附图。

三、 分析说明

Hep G2 细胞株基因组 DNA 扩增后图谱清晰，分型结果良好。

四、 检验结论

1. Hep G2 细胞株 DNA 进行细胞 STR 分型结果显示，细胞株中未发现人类细胞交叉污染。
2. 该细胞株 DNA 分型在细胞库中找到与其细胞分型 98.51%相匹配的细胞株，细胞株名称为 Hep G2。

网站：www.procell.com.cn
电话：400-999-2100
邮箱：techsupport@procell.com.cn
地址：湖北省武汉市高新大道858号生物医药产业园三期C4栋

附表1：细胞株Hep G2的STR位点和Amelogenin位点的基因分型结果。

STR Loci	样品名称：PC-H2024042816	数据库名称：Hep G2
Amelogenin	X, Y	X,Y
CSF1PO	10,11	10,11
D2S1338	19,20	19,20
D3S1358	15,16	15,16
D5S818	11,12	11,12
D7S820	10	10
D8S1179	15,16	15,16
D13S317	9,13	9,13
D16S539	12	12,13
D18S51	13,14	13,14
D19S433	15.2	15.2
D21S11	29,31	29,31
FGA	22,25	22,25
PentaD	9,13	9,13
PentaE	15,20	15,20
TH01	9	9
TPOX	8,9	8,9
vWA	17	17
D6S1043	13	
D12S391	21,25	21,25
D2S441	11.3,14	11.3,14
ExPASy数据库匹配度98.51%，匹配位点数19（https://web.expasy.org/cellosaurus-str-search/）		

备注：

1. **根据国际细胞鉴定委员会(ICLAC)制定的细胞 STR 鉴定标准，细胞系的匹配度≥80%时，认为它们具有相关性，即衍生于共同的祖先细胞；匹配度在 55% 至 80% 之间，需要进一步验证相关性；小于 55%，表明两者不具有相关性。**
2. **图谱有效峰为真实的 PCR 条带;小峰和非特异性条带在计算中忽略不计。**
3. **STR 数据比对结果默认 ExPASy，数据来源包括 ATCC，DSMZ，JCRB 等细胞库以及文献和资料记载，数据库入口 https://web.expasy.org/cellosaurus-str-search/。**

附图1：Hep G2细胞(编号PC-H2024042816)STR位点和Amelogenin位点的基因分型结果。

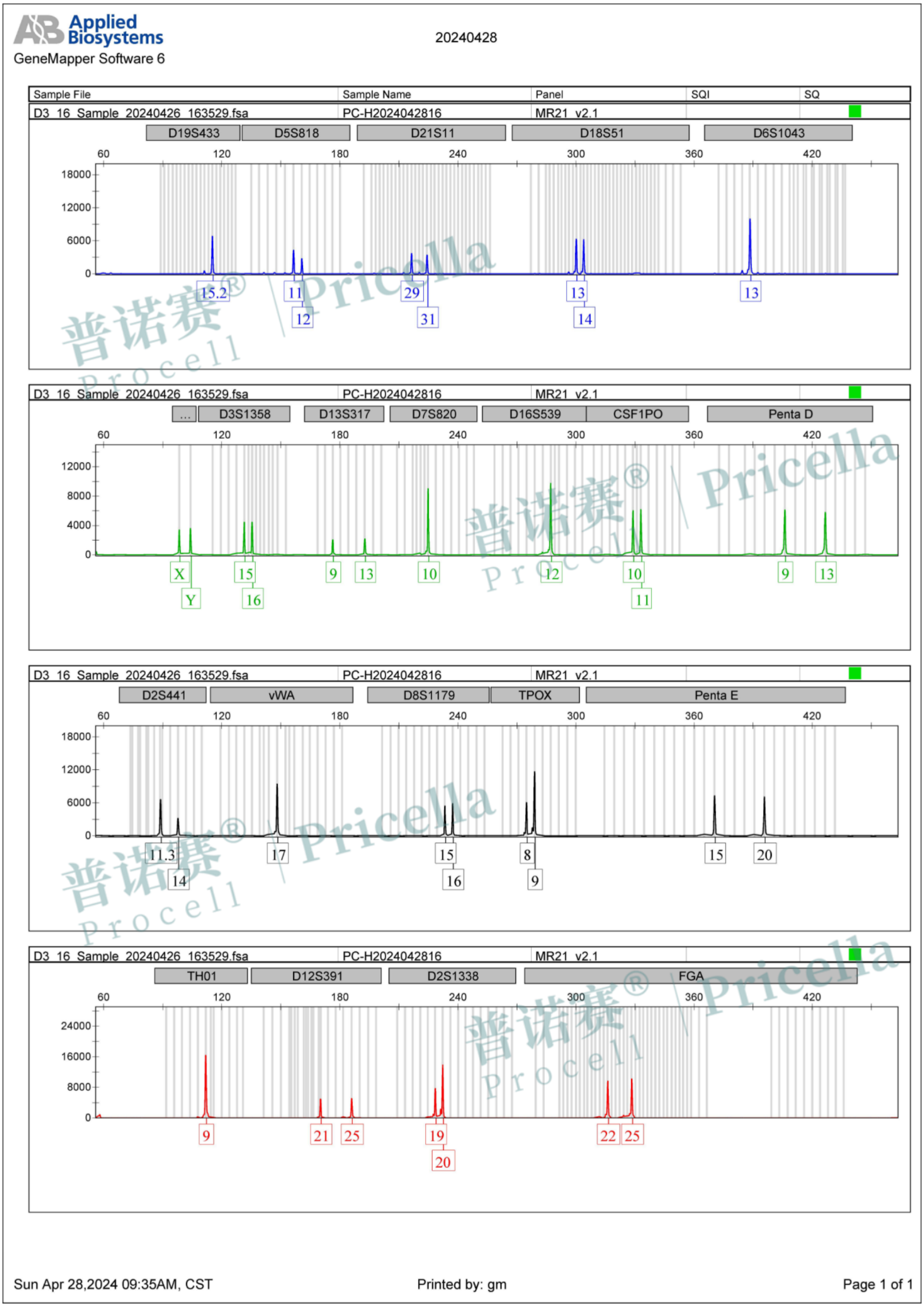

网站：www.procell.com.cn
电话：400-999-2100
邮箱：techsupport@procell.com.cn
地址：湖北省武汉市高新大道858号生物医药产业园三期C4栋

AML12细胞STR鉴定报告

一、 材料处理和检验方法

取适量AML12细胞(编号PC-H2024060622,1×10^6)使用TIANamp Genomic DNA Kit提取DNA，采用小鼠基因分型试剂盒（Mouse STR v2.0）对18个小鼠位点和TH01、D5S818这2个人源位点进行复合扩增，在智阅基因分析仪GenReader 7010上对扩增产物进行检测，使用GeneMapper Software6软件(Applied Biosystems)对检测结果进行分析，并与ExPASy数据库进行比对。

二、 检测结果

实验中阴性及阳性对照结果均正确。

AML12 细胞株的 STR 位点的基因分型结果见附表，分型图谱见附图。

三、 分析说明

AML12 细胞株基因组 DNA 扩增后图谱清晰，分型结果良好。

四、 检验结论

1. AML12 细胞株 DNA 进行小鼠细胞 STR 分型结果显示，分型结果良好，该细胞株为单一来源小鼠细胞，且没有人源细胞污染。
2. 在 ExPASy 细胞库中找到与其细胞分型 100.00%相匹配的细胞，细胞名称为 AML12。

附表1：AML12细胞株PC-H2024060622的STR位点的基因分型结果。

STR Loci	样品名称: PC-H2024060622	数据库名称：AML12
1-1	11	
1-2	13	
2-1	9	
3-2	12	
4-2	20.3	20.3
5-5	14,15	14,15
6-4	16	16
6-7	12	12
7-1	29	
8-1	14,15	
11-2	18,19	
12-1	19	19
13-1	15.1,15.2	
15-3	21.3	21.3
17-2	14,15	
18-3	21	21
19-2	13	
X-1	26	26
TH01		
D5S818		
ExPASy数据库匹配度100.00% ，匹配位点数8（https://web.expasy.org/cellosaurus-str-search/）		

备注：

1. **根据国际细胞鉴定委员会(ICLAC)制定的细胞 STR 鉴定标准，细胞系的匹配度≥80%时，认为它们具有相关性，即衍生于共同的祖先细胞；匹配度在 55% 至 80% 之间，需要进一步验证相关性；小于 55%，表明两者不具有相关性。**
2. **图谱有效峰为真实的 PCR 条带;小峰和非特异性条带在计算中忽略不计。**
3. **STR 数据比对结果默认 ExPASy，数据来源包括 ATCC，DSMZ，JCRB 等细胞库以及文献和资料记载，数据库入口 https://web.expasy.org/cellosaurus-str-search/。**

普诺赛® Procell | Pricella

附图1：AML12细胞（编号为PC-H2024060622）STR位点的基因分型结果。

Applied Biosystems
GeneMapper Software 6

20240523_0603_0606

Sample File	Sample Name	Panel	SQI	SQ
H3 22 Sample 20240618 171259.fsa	PC-H2024060622	MRMouse 18 Kits Panels v1.0		

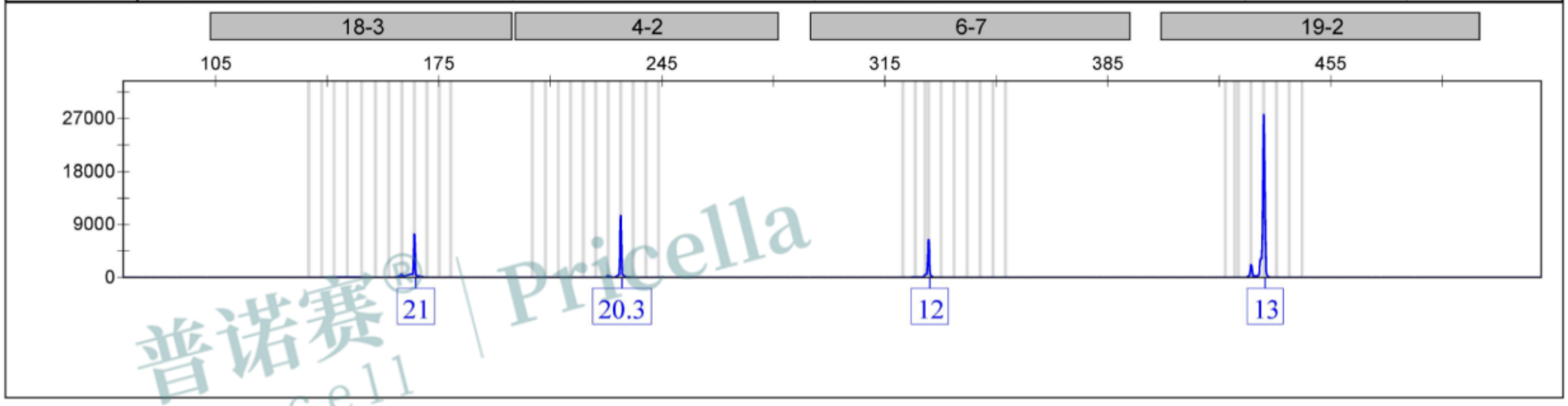

H3 22 Sample 20240618 171259.fsa	PC-H2024060622	MRMouse 18 Kits Panels v1.0		

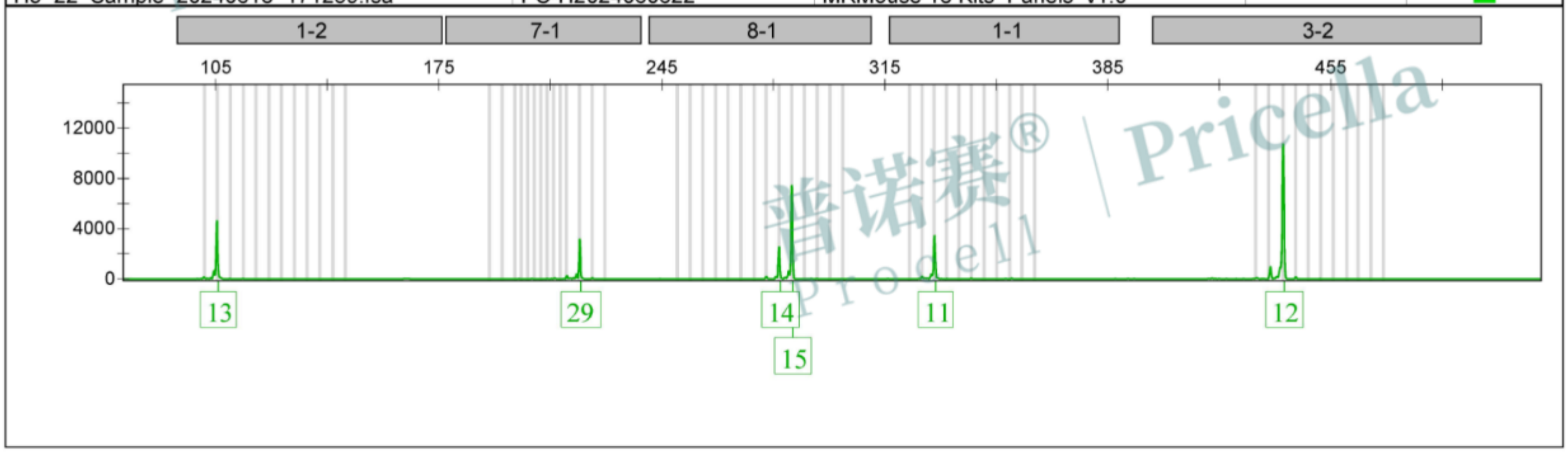

H3 22 Sample 20240618 171259.fsa	PC-H2024060622	MRMouse 18 Kits Panels v1.0		

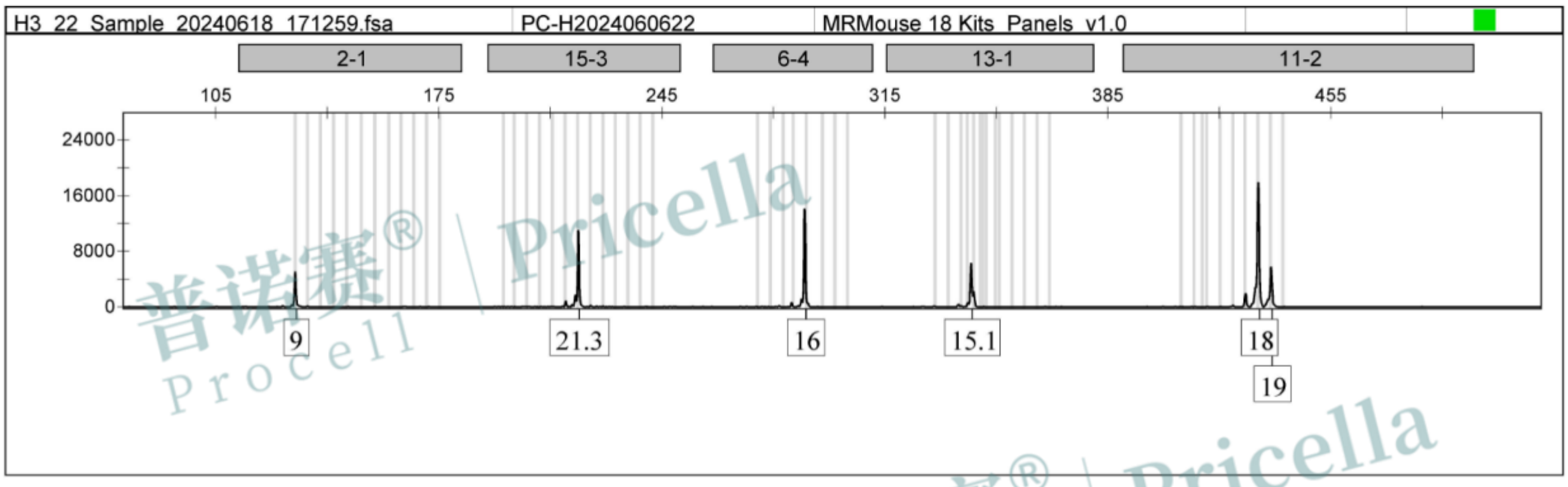

H3 22 Sample 20240618 171259.fsa	PC-H2024060622	MRMouse 18 Kits Panels v1.0		

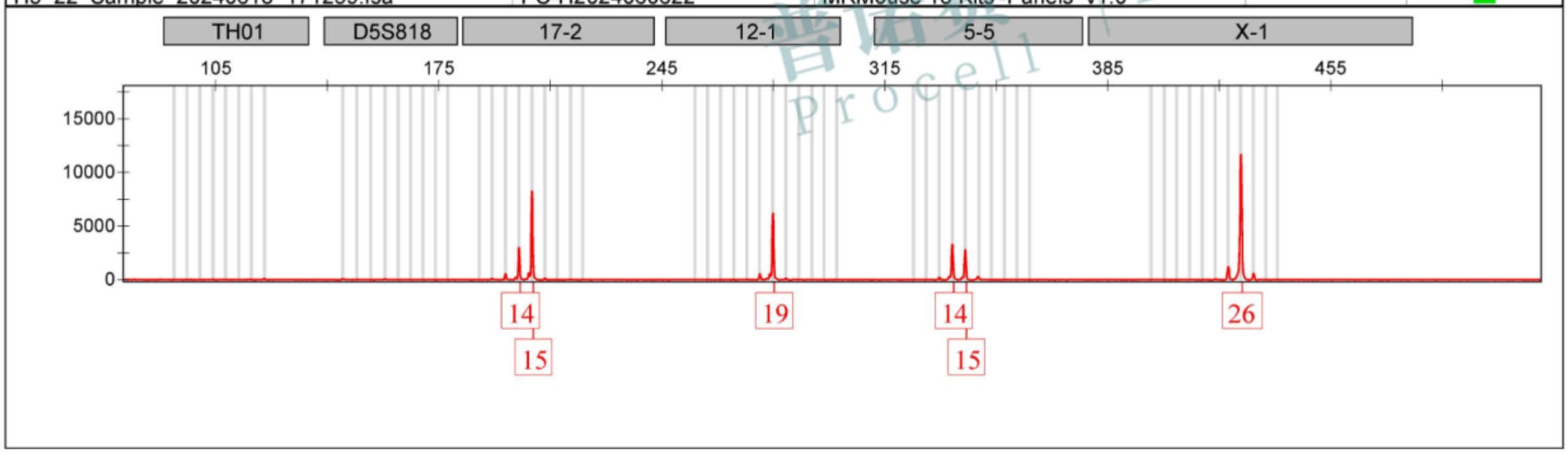

Wed Jun 19,2024 05:50PM, CST   Printed by: gm   Page 1 of 1
