# Supplementary material for: Exertional heat stroke induces liver injury through the activation of the TLR4/NF-κB signaling pathway
Source: Biochem Biophys Rep. 2026 Oct 17;48:102836. doi: 10.1016/j.bbrep.2026.102836 (PMC13696764; doi:10.1016/j.bbrep.2026.102836)
Supplement: Supplementary file 2 [file mmc3.pdf]

Figure 2G. Lane1-3:NC, Lane4-6:EHS, Lane7-9:EHS+TAK-242

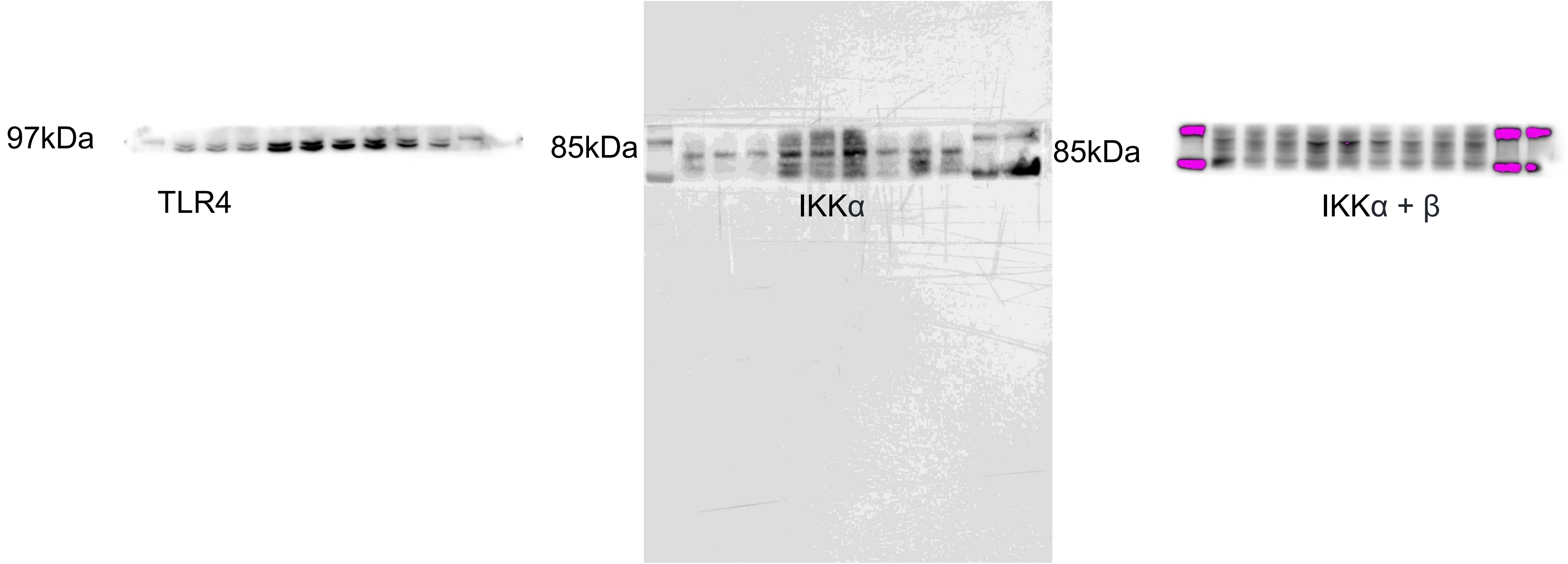

Figure 2G. Lane1-3:NC, Lane4-6:EHS, Lane7-9:EHS+TAK-242

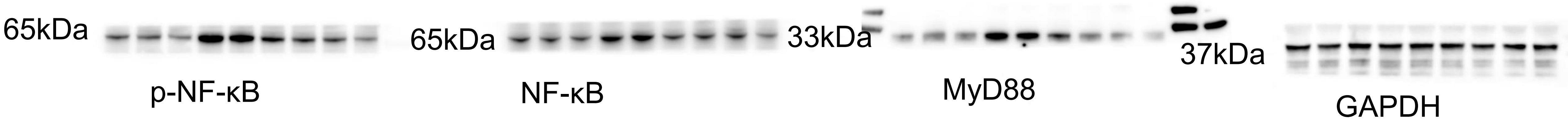

Figure 3C. Lane1: NC, Lane2: TAK-242+HS, Lane3: HS, Lane4: LPS+HS, Lane5: TAK-242+LPS+HS

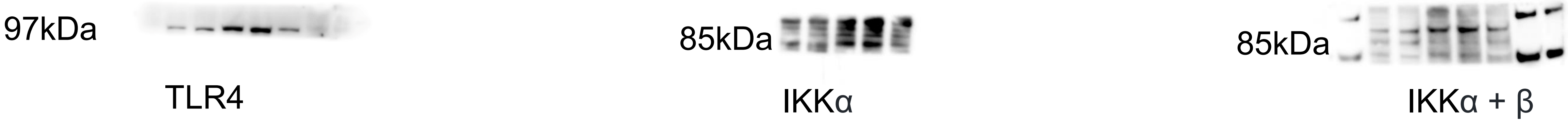

Figure 3C. Lane1: NC, Lane2: TAK-242+HS, Lane3: HS, Lane4: LPS+HS, Lane5: TAK-242+LPS+HS

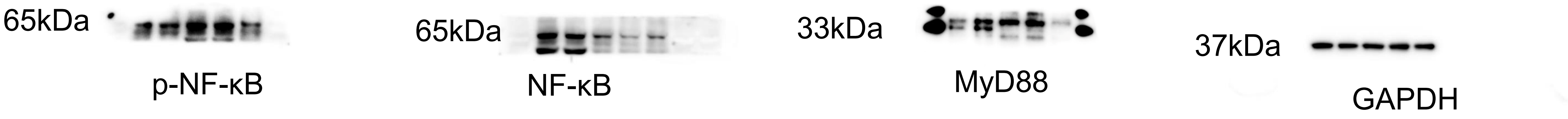

Figure 3D. Lane1: NC, Lane2: TAK-242+HS, Lane3: HS, Lane4: LPS+HS, Lane5: TAK-242+LPS+HS

97kDa 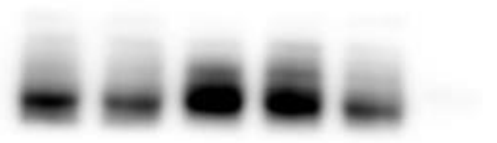

TLR4

85kDa 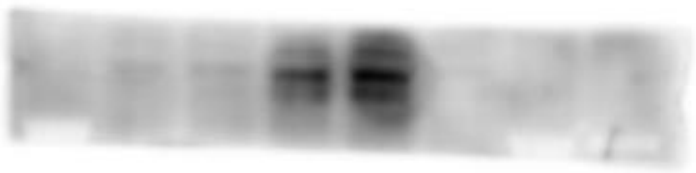

IKKα

85kDa 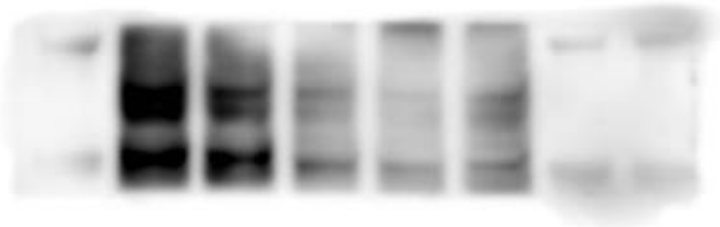

IKKα + β

Figure 3D. Lane1: NC, Lane2: TAK-242+HS, Lane3: HS, Lane4: LPS+HS, Lane5: TAK-242+LPS+HS

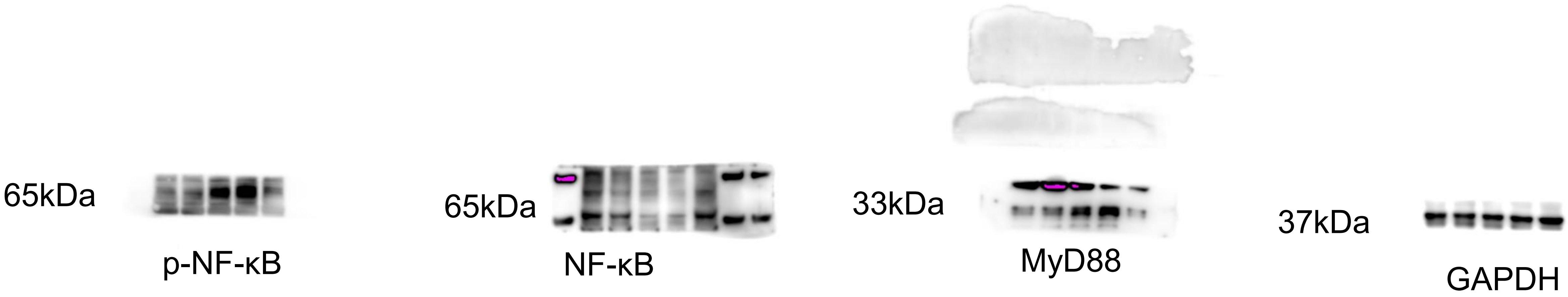

Figure 4D. Lane1-3:NC, Lane4-6:EHS, Lane7-9:EHS+TAK-242

110kDa

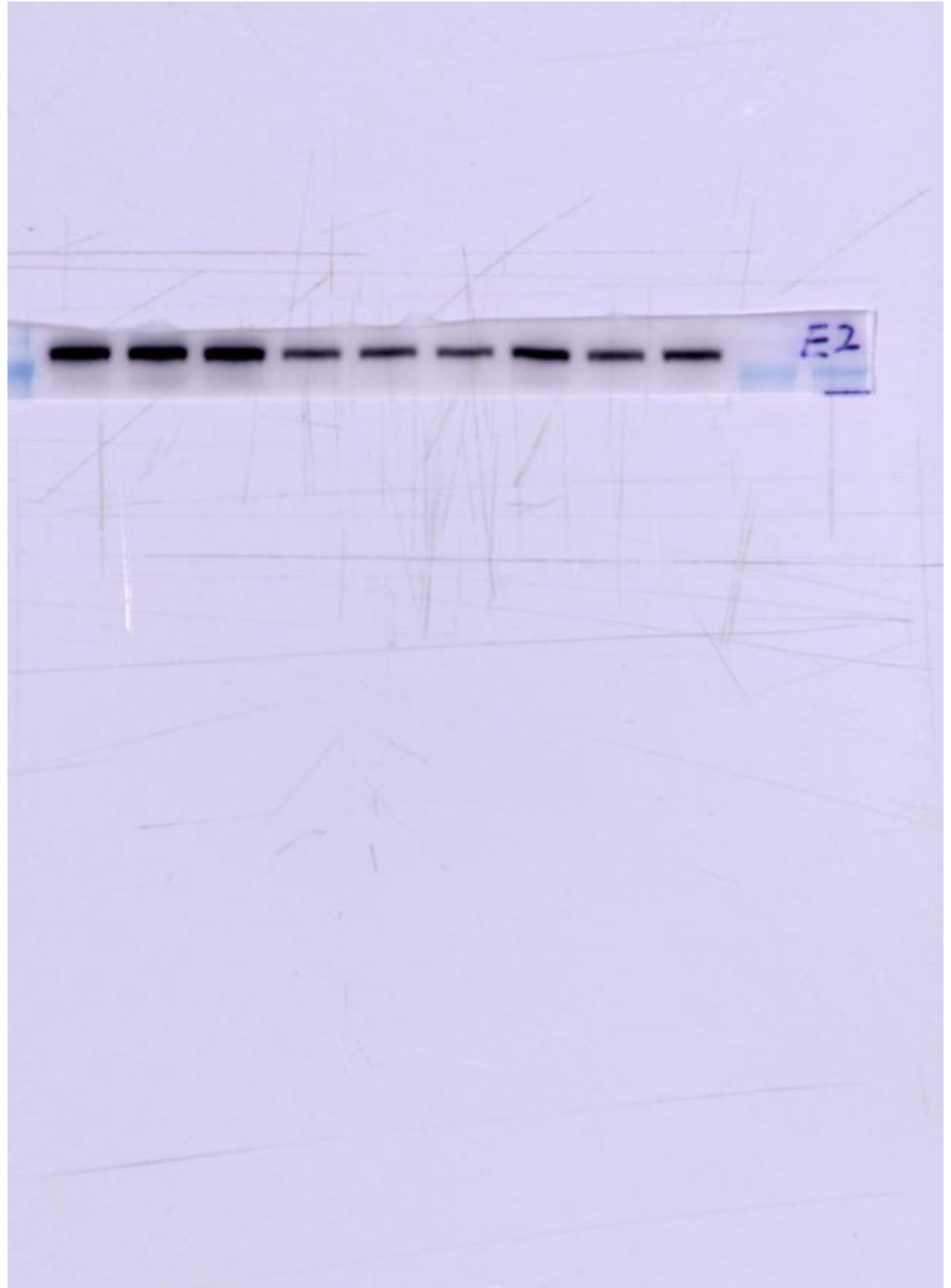

E-cadherin

37kDa

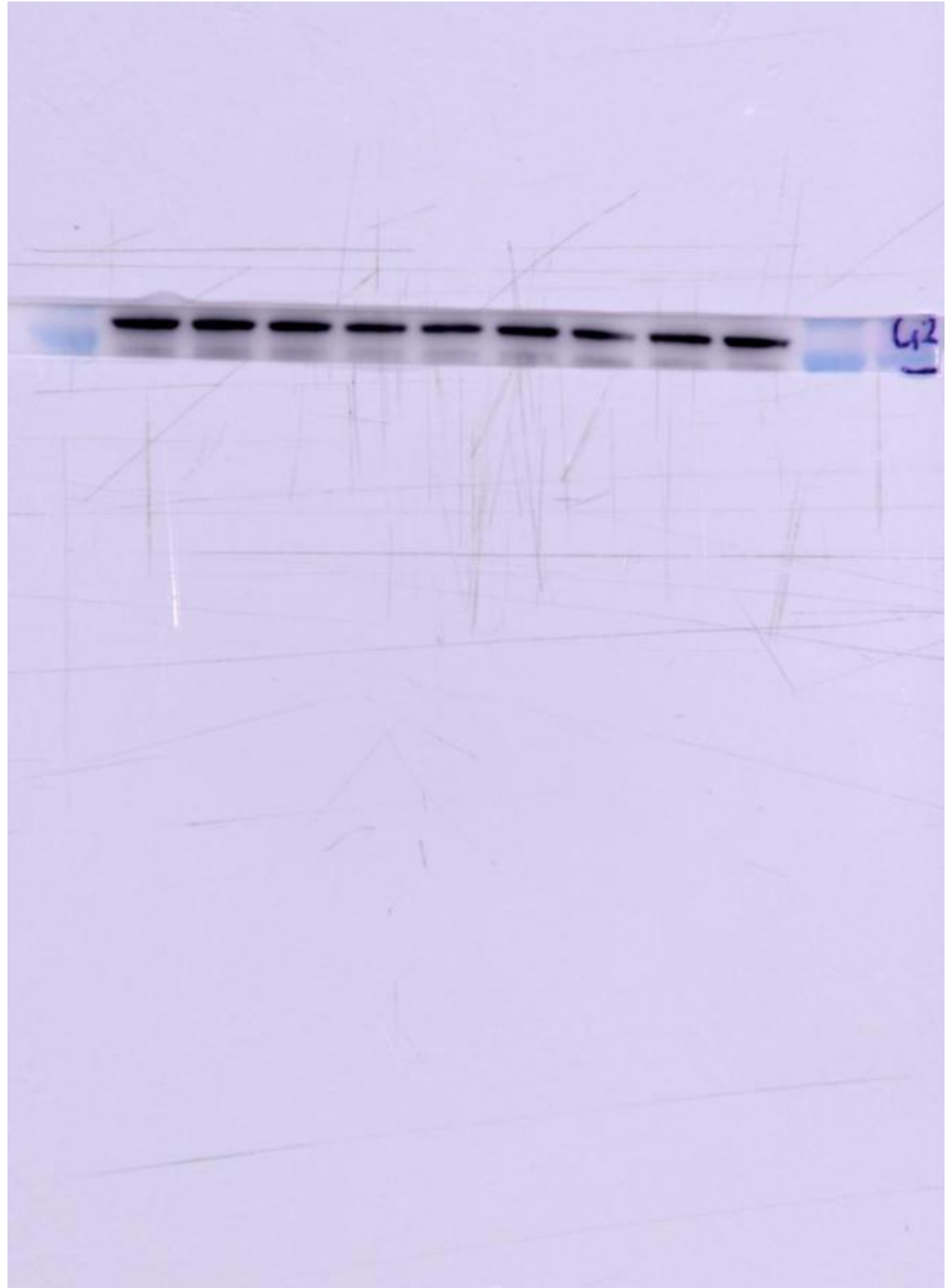

GAPDH

55kDa

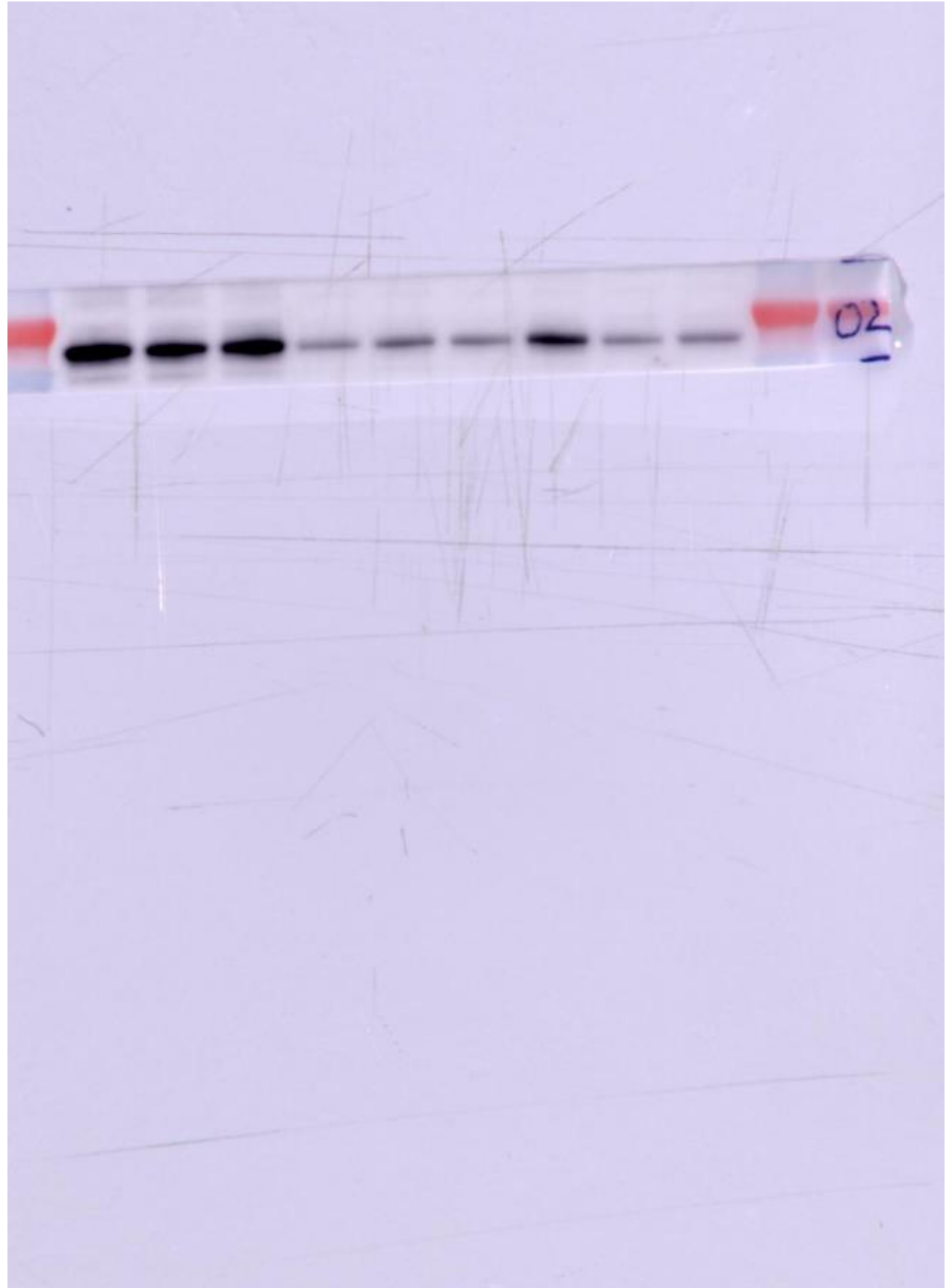

Occludin
