# Supplementary material for: Exertional heat stroke induces liver injury through the activation of the TLR4/NF-κB signaling pathway
Source: Biochem Biophys Rep. 2026 Oct 17;48:102836. doi: 10.1016/j.bbrep.2026.102836 (PMC13696764; doi:10.1016/j.bbrep.2026.102836)
Supplement: Supplementary file 3 [file mmc1.docx]

**Figure S1**. Schematic diagram of core body temperature measurement in rats.

**Figure S2.** TLR4 was detected by immunofluorescence staining in 3 cell climbing slices in each group. And 3 visual fields of each slice were chosen randomly. Data are from three independent experiments and presented as mean ±standard deviation (n=3 per group). **P*<0.05, ****P*<0.001.

Abbreviations: NC, normal control; HS, heat stroke; LPS, lipopolysaccharide; NF-κB, nuclear transcription factor kappa-B.

**Figure S3.** (A, B) Quantitative analysis of TLR4, IKK-α+β, p-NF-κB/NF-κB, and MyD88 protein expression in HepG2 cells (A) and AML-12 cells (B) under the indicated conditions. GAPDH was used as the loading control for TLR4, IKK-α+β, and MyD88. Data are from three independent experiments and are presented as mean ± standard deviation (n=3 per group). **P*<0.05, ***P*<0.01, ****P*<0.001.

Abbreviations: LPS, lipopolysaccharide; NF-κB, nuclear transcription factor kappa-B.

**Figure S4.** Effects of TAK-242 on portal vein lipopolysaccharide (LPS) levels in rats. Data are from three independent experiments and presented as mean ± standard deviation (n=3 per group). ****P*<0.001. NC, normal control; EHS, exertional heat stroke.
